# Unspecific CTL Killing Is Enhanced by High Glucose *via* TNF-Related Apoptosis-Inducing Ligand

**DOI:** 10.3389/fimmu.2022.831680

**Published:** 2022-02-21

**Authors:** Wenjuan Yang, Andreas Denger, Caroline Diener, Frederic Küppers, Leticia Soriano-Baguet, Gertrud Schäfer, Archana K. Yanamandra, Renping Zhao, Arne Knörck, Eva C. Schwarz, Martin Hart, Frank Lammert, Leticia Prates Roma, Dirk Brenner, Grigorios Christidis, Volkhard Helms, Eckart Meese, Markus Hoth, Bin Qu

**Affiliations:** ^1^ Biophysics, Center for Integrative Physiology and Molecular Medicine (CIPMM), School of Medicine, Saarland University, Homburg, Germany; ^2^ Center for Bioinformatics, Saarland University, Saarbrücken, Germany; ^3^ Institute of Human Genetics, School of Medicine, Saarland University, Homburg, Germany; ^4^ Internal Medicine II, University Hospital Saarland, Homburg, Germany; ^5^ Experimental and Molecular Immunology, Department of Infection and Immunity, Luxembourg Institute of Health, Esch-sur-Alzette, Luxembourg; ^6^ Immunology and Genetics, Luxembourg Centre for Systems Biomedicine (LCSB), University of Luxembourg, Belvaux, Luxembourg; ^7^ Faculty of Science, Technology and Medicine, University of Luxembourg, Esch-sur-Alzette, Luxembourg; ^8^ INM-Leibniz Institute for New Materials, Saarbrücken, Germany; ^9^ Hannover Medical School (MHH), Hannover, Germany; ^10^ Odense Research Center for Anaphylaxis, Department of Dermatology and Allergy Center, Odense University Hospital University of Southern Denmark, Odense, Denmark

**Keywords:** High glucose, CTLs, TRAIL, ROS, PI3K-Akt, NFκB

## Abstract

TNF-related apoptosis inducing ligand (TRAIL) is expressed on cytotoxic T lymphocytes (CTLs) and TRAIL is linked to progression of diabetes. However, the impact of high glucose on TRAIL expression and its related killing function in CTLs still remains largely elusive. Here, we report that TRAIL is substantially up-regulated in CTLs in environments with high glucose (HG) both *in vitro* and *in vivo*. Non-mitochondrial reactive oxygen species, NFκB and PI3K/Akt are essential in HG-induced TRAIL upregulation in CTLs. TRAIL^high^ CTLs induce apoptosis of pancreatic beta cell line 1.4E7. Treatment with metformin and vitamin D reduces HG-enhanced expression of TRAIL in CTLs and coherently protects 1.4E7 cells from TRAIL-mediated apoptosis. Our work suggests that HG-induced TRAIL^high^ CTLs might contribute to the destruction of pancreatic beta cells in a hyperglycemia condition.

## Introduction

An elevated level of blood glucose is a typical symptom for diabetes, a metabolic disease. Diabetes affects 422 million people globally and has become the seventh leading cause of death worldwide (1). Diabetes is mainly categorized into two groups, type 1 diabetes and type 2 diabetes. Type 1 diabetes has been identified as an autoimmune disease, for which Cytotoxic T lymphocytes (CTLs) play an important role in destroying the insulin-producing pancreatic beta-cells in an antigen specific manner (2). For type 2 diabetes, CTLs, along with other factors, are reported to be associated with its initiation and progression (3).

CTLs are the key players in the adaptive immune system to destroy pathogen-infected or tumorigenic cells (4). Usually, CTLs employ two major killing mechanisms: lytic granules (LGs) and the Fas/Fas ligand (FasL) pathway. LGs contain cytotoxic proteins, such as the pore-forming protein perforin and the serine proteases termed granzymes. Upon target recognition, LGs are reoriented towards the CTL-target contact site, termed the immunological synapse (IS), and their cytotoxic content will be specifically released into the cleft, resulting in direct lysis or apoptosis of target cells (5). FasL, a member of tumor necrosis factor (TNF) family, is expressed on the surface of CTLs. Engagement of FasL with its Fas receptor, which spans on the target cell surface, initiates apoptotic cascades in target cells (5, 6). Emerging evidence shows that the effector functions of CTLs are highly dependent on their metabolic status (7). Glucose metabolism and the proper access to glucose are essential to maintain CTL effector functions, especially their killing function (8). It is reported that with excessive glucose, calcium influx elicited upon conjugation with target cells is reduced (9) and CTL killing efficiency is elevated partially regulated by Ca^2+^ without affecting lytic granule pathway and FasL expression (10).

TNF-related apoptosis-inducing ligand (TRAIL), similar as FasL, also belongs to the TNF super family. A rich body of studies suggests that TRAIL and/or TRAIL receptors are correlated with progression of diabetes and diabetes-related complications (11, 12). Interestingly, TRAIL is expressed in CTLs (13) to clear viral-infected cells and down-size the effector population to terminate immune responses in an antigen-independent manner (14). TRAIL and TRAIL receptors are linked to both type 1 and type 2 diabetes (15, 16). In humans, four TRAIL receptors are expressed, TRAIL-R1, -R2, -R3 and -R4 (17). The former two (TRAIL-R1 and -R2) are activating receptors, which contain functional death domains to trigger the caspase-8-dependent apoptotic pathway. TRAIL-R3 and -R4 function as decoy receptors, lacking the capability to initiate the death signaling pathway. TRAIL-R1 and/or -R2 are expressed in many normal tissues including pancreas and especially pancreatic beta cells (18, 19).

In this work, we show that TRAIL is substantially up-regulated in high glucose-cultured CTLs and in CTLs from diabetic patients or diabetic mouse models. This high glucose-enhanced TRAIL expression could be diminished by removal of reactive oxygen species (ROS), blockade of NFκB or PI3K/Akt. Pancreatic beta-cells can be killed by TRAIL^high^ CTLs in a TRAIL-dependent manner. Furthermore, we found that treatment of two drugs, metformin and vitamin D, could individually or additively abolish the enhanced TRAIL expression induced by high glucose in CTLs to protect pancreatic beta cells from TRAIL-mediated apoptosis.

## Materials and Methods

### Antibodies and Reagents

All chemicals are from Sigma-Aldrich (highest grade) if not mentioned otherwise. The following antibodies and reagents were purchased from Biolegend: APC/Cy7 anti-human CD3 antibody, BV421 anti-human CD3 antibody, BV421 anti-human CD8 antibody, APC anti-human CD253 (TRAIL) antibody, APC anti-mouse CD253 (TRAIL) Antibody, BV421 anti-mouse CD8a Antibody, PE anti-mouse CD3 Antibody, PerCP anti-human CD25 antibody, APC anti-human CD62L antibody, APC anti-human CD262 (TRAIL-R2), and 7-AAD viability staining solution. The following antibodies were also used: FITC anti-human CD69 (eBiosciences), FITC anti-human CD44 (DAKO), Purified NA/LE mouse anti-human CD253 (BD Biosciences), BV421 mouse anti-human CD263 (TRAIL-R3) (BD Biosciences), Alexa647 mouse anti-human GLUT1 (BD Biosciences), human TRAIL R1/TNFRSF 10A PerCP-conjugated antibody (R&D Systems), and human TRAIL R4/TNFRSF 10D PE-conjugated antibody (R&D Systems). In addition, the following reagents were used: NucView Caspase-3 enzyme substrates (Biotium), Idelalisib (Selleckchem), MK-2206 (Selleckchem), Rapamycin (Selleckchem), Caffeic acid phenethyl ester (CAPE) (R&D Systems), Mitoquinone (MitoQ) (Biotrend), N-acetyl-L-cysteine (NAC) (Merck), vitamin 1,25D3 (Merck), Calcipotriol (TOCRIS), DMSO (Merck), Cellular ROS Assay Kit (Abcam), metformin hydrochloride (Merck), H_2_O_2_ (Merck), and Streptozotocin (Merck).

### Cell Culture

Peripheral blood mononuclear cells (PBMCs) were obtained from healthy donors as described elsewhere (20). Primary human CD8^+^ T cells were negatively isolated from PBMCs using Human CD8^+^ T Cell isolation Kits (Miltenyi Biotec). Human CD8^+^ T cells were stimulated with CD3/CD28 activator beads (Thermo Fisher Scientific) and cultured in DMEM medium (Thermo Fisher Scientific) containing normal (5.6 mM) or high glucose (25 mM) for up to three days if not otherwise mentioned. Levels of glucose in medium was examined every day using “Contour Next Sensoren” test strips (SMS Medipool) and consumed glucose was compensated accordingly. If CTLs were cultured longer than three days, human recombinant IL-2 (Miltenyi Biotec) was added to the medium every two days from day 2 on (100 U/ml). All cells were cultured at 37°C with 5% CO_2_. Human pancreatic beta cell line 1.4E7 was purchased from Merck and cultured in RPMI-1640 medium (Thermo Fisher Scientific) supplemented with 2 mM glutamine, 1% Penicillin-Streptomycin plus 10% FCS (ThermoFisher Scientific).

### Flow Cytometry Analysis

For cell surface staining, cells were washed twice with PBS/0.5% BSA and stained for 30 minutes at 4°C in dark using corresponding antibodies mentioned in the figure legends. For intracellular staining, cells were fixed in pre-chilled 4% PFA and permeabilized with 0.1% saponin in PBS containing 5% FCS and 0.5% BSA, followed by the immunostaining as described above. Flow cytometry data were acquired using a FACSVerse flow cytometer (BD Biosciences) and were analyzed with FlowJo v10 (FLOWOJO, LLC).

### Assays for Apoptosis and Viability

To assess cell apoptosis, 1.4E7 cell were co-cultured with primary human CD8^+^ T cells and incubated at 37°C with 5% CO_2_. Cells were harvested at various time points as indicated in the text and stained with BV421-CD3, and then incubated with NucView Caspase-3 Substrates at room temperature for 30 minutes, followed with analysis using flow cytometry. To determine the viability of CTLs, cells were stained with 7-AAD.

### Diabetic Mouse Model

C57BL/6N mice were injected with streptozotocin intraperitoneally for five days consecutively (50 mg/kg per day) and were sacrificed at day 21. Blood samples were taken from the tail vein and glucose level was tested by standard test strips every day after injection. Mice with blood glucose level more than 250 mg/dL one week after the first injection were considered as diabetic.

### Microarray and Analysis

For transcriptome analyses, total RNA of CD8^+^ human T cells was extracted by miRNeasy Mini Kit (Qiagen), following the manufacturers’ instructions, and quantified using NanoDrop 2000c Spectrophotometer (Thermo Fisher Scientific). The quality of the RNA samples was assessed by determining the corresponding RIN (RNA integrity number) values. For this purpose, an Agilent 2100 Bioanalyzer instrument was used together with the RNA 6000 Nano assay from Agilent Technologies (Santa Clara). An excellent quality of the analyzed total RNA was verified by RIN values of 10 for all samples. To determine the cellular transcriptomes, 100 ng of the total RNA was analysed by microarray. The microarray analyses were performed as previously described (21). Differential expression analysis of the Agilent microarray data was performed with the Linear Models for Microarray Data (limma) R package (22). First, samples were corrected for background (NormExp) and quantile normalized, respectively. Control probes used for background correction, probes without associated gene symbols, and genes classified as not expressed in at least 6 out of 12 arrays by the Agilent feature extraction software were removed from the dataset. After the filtering step, the dataset contained a total of 29,037 transcripts, corresponding to 19,834 genes. For differential expression analysis, a linear model was fitted to each individual sample. Then, a linear model was calculated between samples in high glucose (HG) or normal glucose (NG) conditions. Differentially expressed genes were identified with a t-test. The associated p-values were calculated for each gene with an empirical Bayes method and adjusted for multiple testing with the Benjamini-Hochberg method (23). A gene was classified as differentially expressed (DE) for a given contrast if its adjusted p-value was below 0.05. Among the DE genes, an enrichment analysis for gene annotations was performed with the limma package. Also, an enrichment analysis based on protein interaction subnetworks was carried out by the pathfindR package (24).

### Seahorse Assay

Primary human CD8^+^ T cells were stimulated either in normal (5.6 mM) or high glucose (25 mM) for three days. At day 3, CTLs were counted and seeded in 96-well XF Cell Culture Microplate in XF Seahorse DMEM medium at a cell concentration of 3×10^5^ cells/well. Following the manufacturer’s instructions (Agilent), the extracellular acidification rate (ECAR) and oxygen consumption rate (OCR) were measured using the XF Glycolytic Stress Test and XF Cell Mitochondrial Stress Test kits, respectively.

### Patient Materials

Blood samples were collected from patients with diabetes type 1 and 2 (using the current diagnostic criteria from the American Diabetes Association-ADA) and healthy control subjects (all with normal glycated haemoglobin level (HbA1c < 5,7%) at the day of the blood sample collection). Both patients and healthy controls were recruited in the Department of Internal Medicine II in the Saarland University Medical Center, Homburg, Saarland, Germany. Written informed consent was obtained from all subjects before blood sampling, strictly following the procedure described in the ethical approval of the ethic committee of the medical association of Saarland (Ethic Vote Nr: Ha 84/19). PBMCs were obtained from the blood samples and were stimulated with CD3/CD28 activator beads (Thermo Fisher Scientific) and cultured in DMEM containing normal (5.6 mM) or high glucose (25 mM) for three days, supplemented with recombinant IL-2 (100 U/ml, Miltenyi).

### Intracellular ROS Detection

Cellular ROS Assay Kit (Abcam) was used to determine intracellular ROS. Briefly, at 6 hours after activation of CD3/CD28 activator beads, the CTLs were stained with BV421-CD8 for 30 minutes at 4°C in dark, and then incubated with dichlorofluorescein diacetate (DCFDA, 20 µM) at 37°C for 30 minutes, followed by analysis using flow cytometry.

### Quantitative RT-PCR

The mRNA expression analysis was carried out as described before (25). Briefly, total RNA was isolated from CTLs using TRIzol reagent (ThermoFisher Scientific). Then the isolated RNA was reversely transcribed into complementary DNA (cDNA) and relative gene expression was performed by qRT-PCR using CFX96Real-TimeSystemC1000 Thermal Cycler (Bio-Rad Laboratories). TATA box-binding protein (TBP) was used as the housekeeping gene for the normalization of the target genes. Primer sequences are as follows (forward/reverse): TBP (5’-CGGAGAGTTCTGGGATTGT-3’/5’-GGTTCGTGGCTCTCTTATC-3’). Pre-designed primers were purchased for TRAIL (QT00068957) and Glut1 (QT00079212).

### Statistical Analysis

Data are presented as mean ± SD. GraphPad Prism Software (San Diego, CA, USA) was used for statistical analysis. The differences between two groups were analyzed by the Student’s t-test. For multiple comparisons, two-way ANOVA or one-way ANOVA was performed followed by Bonferroni test.

## Results

### Expression of TRAIL Is Up-Regulated in CTLs by High Glucose and TRAIL^high^ CTLs Induces Destruction of Pancreatic Beta Cells

**Figure 1:**
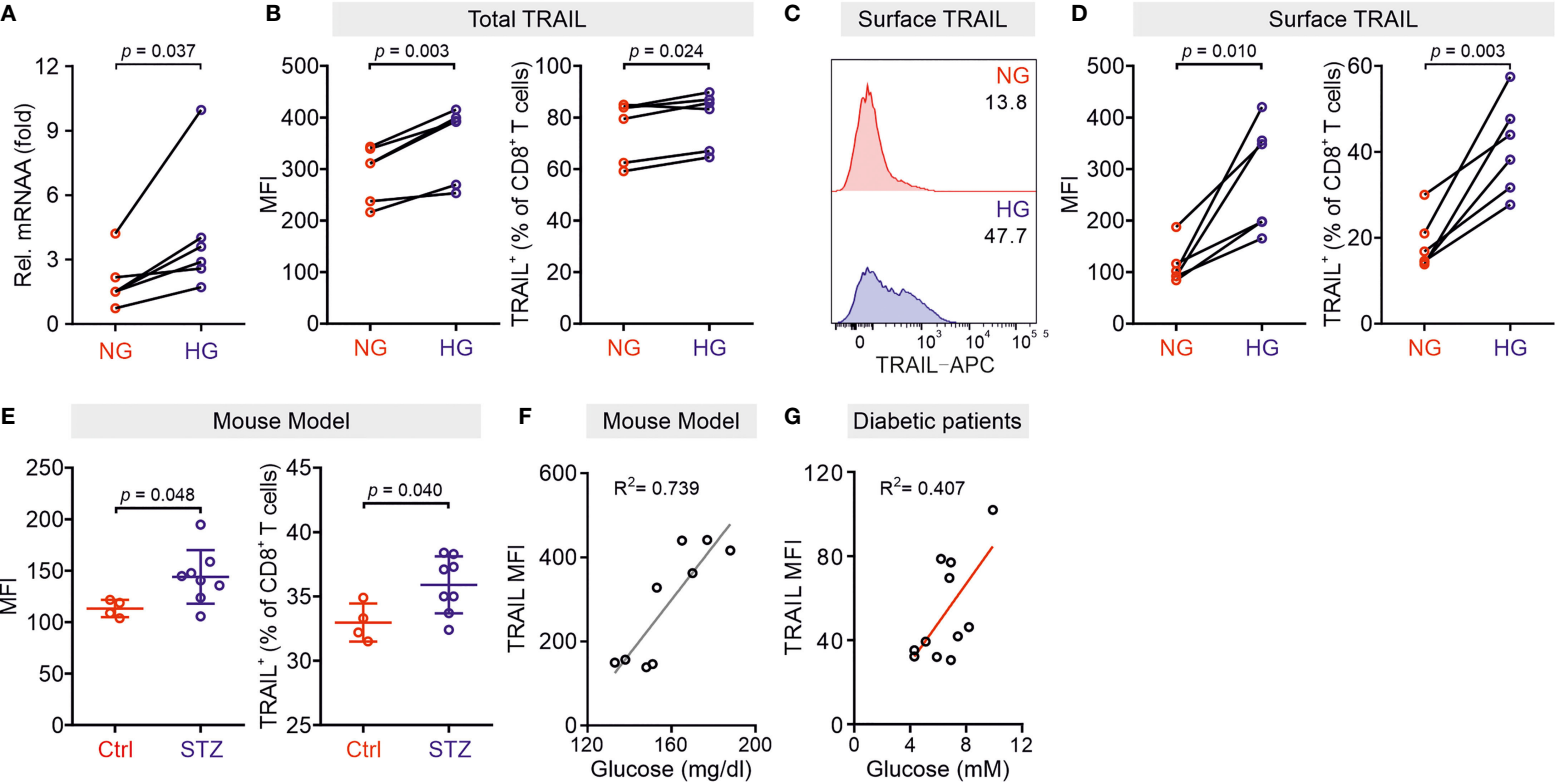
TRAIL is up-regulated in CTLs in environments with high glucose. Primary human CD8^+^ T cells were stimulated with CD3/CD28 beads for 3 days in NG (5.6 mM) or HG (25 mM) medium. **(A)** Relative mRNA expression levels of TRAIL in CTLs were quantified by qRT-PCR (n = 6 donors from three independent experiments). **(B–D)** Expression of TRAIL in CTLs was detected by flow cytometry. CTLs were stained with the antibodies against CD8 and TRAIL following permeabilization **(B)** or without permeabilization **(C, D)** for total or surface protein level of TRAIL (n = 6 donors from three independent experiments). **(E, F)** TRAIL is up-regulated in diabetic mice. Mouse splenocytes were isolated and stained with PE-mCD3, BV421-mCD8, and APC-mTRAIL for analysis using flow cytometry (Ctrl = 4, STZ mice = 8). Data are represented as Mean ± SD. Correlation of TRAIL expression in CD8^+^ T cells and blood glucose is shown in **(F, G)** Positive correlation of TRAIL expression in freshly isolated CD8^+^ T cells from 11 diabetic patients with the blood glucose levels from ten independent experiments. MFI, mean fluorescent intensity. Data were analyzed by two-tailed paired Student’s *t* test **(A, B, D)**, two-tailed unpaired Student’s *t* test **(E)**, trendline analysis **(F)**, or Pearson’s correlation coefficient analysis **(G)**. Connected lines are the data from the same donor.

Our previous work shows that culturing CTLs in high glucose-containing medium does not alter their expression of perforin, granzymes, FasL, and degranulation (10). Thus, in this study we analyzed the impact of high glucose on expression of TRAIL in CTLs. We used negatively isolated primary human CD8^+^ T cells from healthy donors and stimulated the cells with CD3/CD28 antibody-coated beads in presence of normal (5.6 mM, NG) or high glucose (25 mM, HG) for three days (hereafter referred to as NG or HG-CTLs). At mRNA level, TRAIL was substantially up-regulated in HG-CTLs compared to NG-CTLs (**Figure 1A**). Concomitantly, at protein level, not only the total expression of TRAIL was considerably enhanced (**Figure 1B**), but also the expression of TRAIL on the surface was significantly elevated in HG-CTLs compared to NG-CTLs (**Figures 1C, D**). To test the *in vivo* relevance of this finding, we used a streptozotocin-induced diabetic mouse model. Compared to CTLs from the control group, CTLs from diabetic mice exhibited significantly elevated levels of TRAIL (**Figure 1E**). The level of blood glucose was positively correlated with expression of TRAIL (**Figure 1F**). Furthermore, we examined TRAIL expression in freshly isolated CD8^+^ T cells from diabetic patients, which was also positively correlated with the level of blood glucose (**Figure 1G**). These findings suggest that TRAIL expression in CTLs is up-regulated by HG and that glucose levels correlate with TRAIL expression *in vivo* in the context of diabetes.

**Figure 2:**
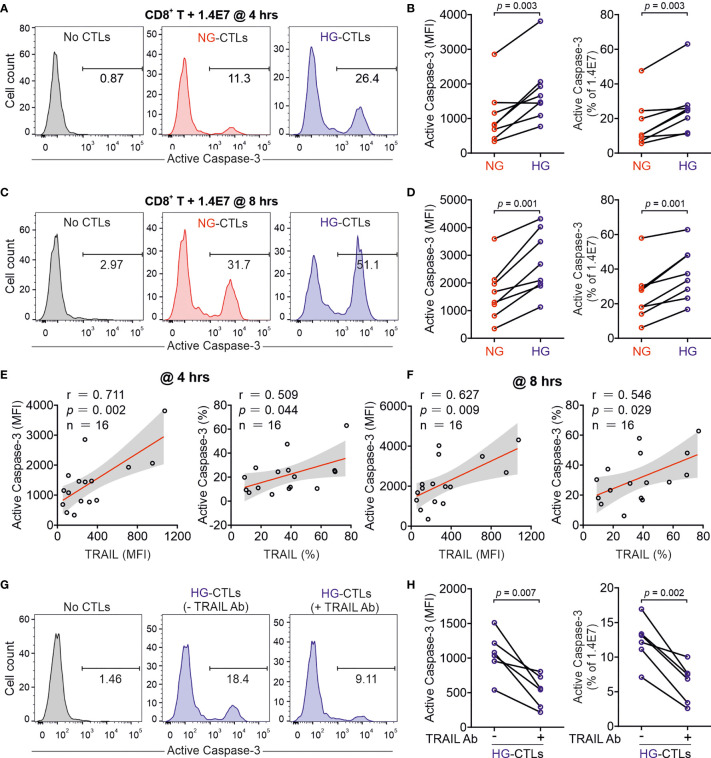
Up-regulation of TRAIL in HG-CTLs enhances apoptosis of pancreatic beta cells. Primary human CD8^+^ T cells were stimulated with CD3/CD28 beads for 3 days in NG (5.6 mM) or HG (25 mM) medium. **(A–D)** Apoptosis of pancreatic beta cells induced by CTLs were measured by staining active Caspase-3 using flow cytometry. Human pancreatic beta cells 1.4E7 were incubated with CTLs with an effector to target (E:T) ratio of 20:1 for 4 hours **(A, B)** or 8 hours **(C, D)** (n = 8 donors from five independent experiments). One representative donor out of eight is shown in **(A, C)** respectively. **(E, F)** Correlation of expression of TRAIL in CD8^+^ T cells with apoptosis of 1.4E7 cells (Caspase-3 activity) (n = 16 donors from five independent experiments). **(G, H)** CD8^+^ T cells-induced beta cell apoptosis is TRAIL dependent. Caspase-3 activity was analyzed in co-culture of 1.4E7 cells and CTLs in the presence or absence of anti-human TRAIL antibody (50 µg/ml) for 4 hours. One representative donor is shown in **(G)** and the quantification is shown in **(H)** (n = 6 donors from two independent experiments). MFI, mean fluorescent intensity. Data were analyzed by two-tailed paired Student’s *t* test **(B, D, H)** or Pearson’s correlation coefficients **(E, F)**. Connected lines are the data from the same donor.

Given the fact that treatment of soluble TRAIL can induce apoptosis of human pancreatic beta cells (19), we then examined whether CTLs expressing TRAIL could have a similar effect. We used the human pancreatic beta cell line 1.4E7, which is a hybrid cell line derived from electrofusion of primary human pancreatic islets with a human pancreatic ductal carcinoma cell line PANC-1. We incubated 1.4E7 cells with CTLs and used activity of caspase-3 in 1.4E7 cells as a readout for CTL-induced apoptosis. The results show that HG-cultured CTLs exhibited significantly higher killing capacity compared to their counterparts in NG (**Figures 2A–D** and **Figures S1A, B**). Of note, apoptosis of beta cells is positively correlated with TRAIL expression (**Figures 2E, F** and **Figure S1C**). To test a potential causal relation between TRAIL expression and CTL cytotoxicity against beta cells, we blocked TRAIL function with its neutralizing antibody. The analysis of caspase-3 activity shows that TRAIL blockade diminished CTL-mediated killing against 1.4E7 beta cells to a large extent for HG-CTLs (**Figures 2G, H**). Next, we examined the expression of TRAIL receptors on 1.4E7 cell surface. We found that out of four TRAIL receptors, TRAIL-R2 was predominantly expressed (**Figure S1D**), which likely mediates TRAIL^high^ CTL-induced apoptosis of 1.4E7 cells. Taken together, our results indicate that HG-CTLs are able to destroy TRAIL-R2^high^ pancreatic beta cells in a TRAIL-mediated manner.

### Glucose Metabolic Processes in CTLs Are Enhanced by High Glucose

**Figure 3:**
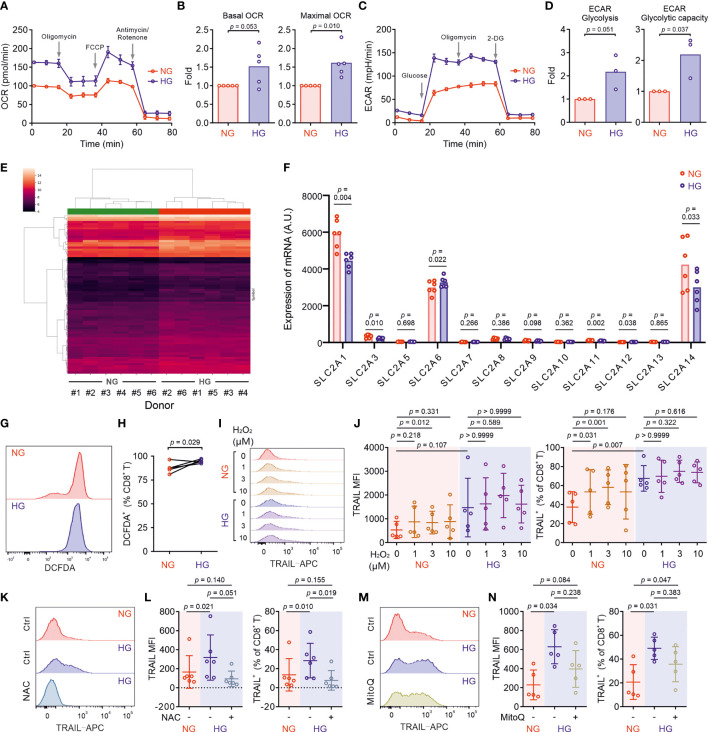
Metabolic processes in CTLs are reprogrammed by high glucose. Primary human CD8^+^ T cells were stimulated with CD3/CD28 beads for 3 days in NG (5.6 mM) or HG (25 mM) medium. **(A–D)** Oxidative phosphorylation (n = 5 donors) and glycolysis (n = 3 donors) of CTLs from two independent experiments were determined with seahorse assay. One representative donor for oxidative phosphorylation and glycolysis is shown in **(A, C)**, respectively. **(E)** Heatmap of log2-transformed gene expression data. The microarray data was normalized using quantile normalization. Duplicate probes for genes were aggregated by taking the median intensity. Genes were filtered for those with an absolute fold change > 1.5, and a Benjamini-Hochberg adjusted *p*-value < 0.05. **(F)** Expression of glucose transporters at mRNA level from the transcriptomics data (**Table S1**) of 6 donors collected from five independent stimulations and two independent microarray analyses. A.U. stands for arbitrary units. **(G, H)** ROS production in CD8^+^ T cells was determined at 6 hours after CD3/CD28 bead stimulation by DCFDA (n = 5 donors from three independent experiments). One representative donor is shown in **(G)** Connected lines in **(H)** are the data from the same donor. **(I, J)** H_2_O_2_ enhances TRAIL expression in CTLs in NG. CD8^+^ T cells were stimulated with CD3/CD28 beads in presence or absence of H_2_O_2_ for 3 days (n = 5 from three independent experiments). One representative donor is shown in **(I, K–N)** Inhibition of ROS production abolishes HG-enhanced TRAIL expression in CTLs. NAC (**K**, **L**, 10 mM, n = 6 donors) or MitoQ (**M, N**, 0.4 μM, n = 5 donors) from three independent experiments was added during the activation for 3 days. One representative donor for NAC and MitoQ is shown in **(K, M)**, respectively. Results are represented as Mean ± SD. Data were analyzed by two-tailed unpaired Student’s *t* test **(B, D)**, two-tailed paired Student’s *t* test **(H)** or one-way ANOVA with Bonferroni’s multiple comparison test **(J, L, N)**.

Glucose metabolism encompasses the intracellular biochemical processes to breakdown and utilize glucose to generate energy, including oxidative phosphorylation and glycolysis. Using the Seahorse assay, we evaluated the oxygen consumption rate (OCR) and the extracellular acidification rate (ECAR). We found that HG-cultured CTLs exhibited significantly elevated OCR and ECAR compared to their counterparts cultured in NG (**Figures 3A–D**).

Given the essential role of mTOR in regulating glycolysis in T cells (26), we next examined the possible involvement of mTOR in HG-induced metabolic reprogramming in CTLs. To our surprise, no difference was identified between NG- and HG-CTLs regarding mTOR activation (**Figure S2A**). Since glucose uptake and transport play an essential role for overall glucose metabolism, we examined the expression of glucose transporter 1 (Glut1) and found that although its expression was down-regulated at mRNA level in HG-CTLs (**Figure S2B**), at the protein level it remained unchanged (**Figures S2C, D**). In addition, the fraction of Glut1 transported to the plasma membrane was also not altered by HG (**Figures S2E, F**). Together, our results suggest that neither mTOR nor glucose transport are likely to be responsible for HG-reprogrammed glucose metabolic processes in CTLs.

To gain deeper insights into the genes affected by HG for glucose metabolic reprogramming, we compared transcriptomes of NG-CTLs and HG-CTLs (**Figure 3E** and **Table S1**). First, we examined the expression of all glucose transporters. We found that three glucose transporters (Glut1/SLC2A1, Glut3/SLC2A3, and Glut14/SLC2A14) were predominantly expressed in CTLs, and moderate downregulation in Glut1 was observed in HG-CTLs compared to their NG counterparts (**Figure 3F**), which is in a good agreement with our data from quantitative PCR (**Figure S2B**). No further difference in expression of glucose transporters was identified between NG- and HG-CTLs (**Figure 3F**), further supporting our conclusion that glucose transport is unlikely to be responsible for HG-reprogrammed metabolic processes in CTLs. With regards to enriched GO terms, the most significant change happened in the cellular metabolism. When comparing HG- to NG-CTL samples, many genes involved in metabolic processes are significantly changed with a total of 58 being significantly up-regulated and 47 genes being significantly down-regulated (**Table S2**, Row 2), which are associated with a total of 265 GO annotations describing various metabolic processes (**Table S2**). Furthermore, 6 genes annotated with the metabolism of ROS were significantly deregulated (**Table S3**).

We then examined whether ROS was involved in HG-enhanced TRAIL expression. We first determined ROS production in CTLs using DCFDA, a fluorescent probe used to detect ROS in living cells (27). We found that upon activation, ROS produced in HG-CTLs was higher than that in their NG counterparts (**Figures 3G, H**). To explore whether ROS is involved in HG-enhanced TRAIL expression, we first added H_2_O_2_, a relatively stable form of ROS, during NG- and HG-culture. We found that in NG condition, compared to the control group, with 1 µM H_2_O_2_, the fraction of TRAIL^+^ CTLs was enhanced (p=0.031) and the mean of MFI is also higher but statistically not significant (p=0.218); with 3 µM H_2_O_2_, both the fraction of TRAIL^+^ CTLs and MFI were enhanced (p=0.001/0.012); whereas with 10 µM H_2_O_2_, the mean of the fraction of TRAIL^+^ CTLs and MFI were enhanced but statistically not significant (p=0.176/0.331) (**Figures 3I, J**). While in HG-CTLs, neither MFI nor the fraction of TRAIL^+^ CTLs were altered by addition of H_2_O_2_ (**Figures 3I, J**). This implies that the elevation in TRAIL expression by HG is likely *via* enhanced ROS in HG-CTLs. To test this hypothesis, we used ROS scavengers either with a general inhibition effect [N-acetyl-L-cysteine (NAC)] or specifically targeted to mitochondrial ROS (MitoQ). We found that when ROS production in cytosol was removed by NAC, the expression of TRAIL was drastically decreased in HG-CTLs to a comparable level as in NG-CTLs (**Figures 3K, L**). In contrast, removal of mitochondria-produced ROS by MitoQ in HG-CTLs did not significantly alter TRAIL expression (**Figures 3M, N**). Thus, our results suggest that in CTLs, ROS, especially cytosolic ROS, plays a key role in HG-enhanced TRAIL expression.

### HG-Enhanced TRAIL Expression on CTLs Is Regulated by PI3K/Akt and NFκB

**Figure 4:**
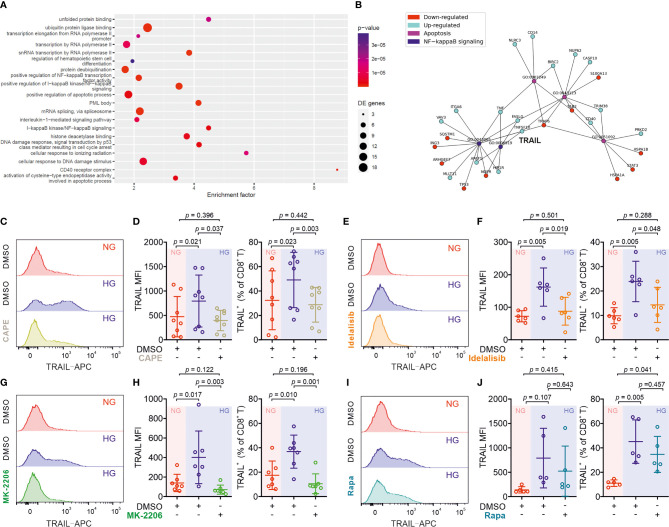
Up-regulation of TRAIL in CTLs is regulated by the PI3K/Akt/NFκB axis. **(A)** Enriched KEGG pathways for the comparison between HG and NG conditions. **(B)** Ten most significantly enriched/depleted Gene ontology terms in HG vs NG conditions. Enrichment analysis was performed with the *pathfindR* package, which creates a sub-network of the human protein-protein interaction network that only contains genes with significant differential expression. The enrichment factor of an annotation is calculated as the fraction of proteins having this annotation in the sub-network, relative to the entire network. **(C–J)** Primary human CD8^+^ T cells were stimulated with CD3/CD28 beads for 3 days in presence or absence of NF-κB specific inhibitor CAPE (**C, D**, 5 μM, n = 8 donors from three independent experiments), PI3K inhibitor Idelalisib (**E**, **F**, 300 nM, n = 6 donors from three independent experiments), Akt inhibitor MK-2206 (**G, H**, 200 nM, n = 7 donors from four independent experiments), or mTOR inhibitor Rapamycin (**I, J,** 600 nM, n = 5 donors from three independent experiments). CD8^+^ T cells were stained with antibody against CD8 and TRAIL and were analyzed by flow cytometry. Representative donors are shown in **(C, E, G, I)**. Data are represented as Mean ± SD and *p* values were assessed by one-way ANOVA with Bonferroni’s multiple comparison test.

We then analyzed KEGG pathways based on the transcriptomics data (**Figure 4A**). This analysis also revealed a statistically significant up-regulation of 11 genes including TRAIL (synonym name TNFSF10) annotated with the KEGG pathway *apoptosis* in HG samples (**Table S4**). Among the top 10 GO terms affected by HG, TRAIL is linked both to “positive regulation of IκB kinase/NFκB signaling” and to “positive regulation of apoptotic process” (**Figure 4B**).

We then focused on the involvement of NFκB in HG-enhanced TRAIL expression and tested the effect of the NFκB specific inhibitor CAPE. Notably, the expression of TRAIL on HG-cultured CTLs treated with CAPE was substantially reduced compared to untreated control cells (**Figures 4C, D**). This indicates that NFκB is indispensable for expression of TRAIL in CTLs. NFκB function can be regulated by the PI3K-Akt pathway, which was among the deregulated KEGG pathways (**Figure 4A**). We therefore used the PI3Kδ inhibitor idelalisib to block the activity of PI3K. We found that in HG-CTLs, abruption of PI3K function with the corresponding inhibitor reduced the expression of TRAIL to the level of NG (**Figures 4E, F**). We further examined Akt, a molecule downstream of PI3K and upstream of NFκB. We found that disruption of Akt function by MK-2206 abolished HG-enhanced TRAIL expression (**Figures 4G, H**). Apart from NFκB, mTOR is also regulated by Akt (28). To examine whether mTOR is involved in HG-enhanced TRAIL expression, we used rapamycin, a specific inhibitor for mTOR, to functionally block mTOR activity. We found that in HG-CTLs even with the highest concentration (600 nM) of rapamycin, the change in TRAIL expression was not statistically significant, and the fraction of TRAIL^+^ CTLs in rapamycin-treated HG-CTLs remained significantly higher than the level in NG-CTLs (**Figures 4I, J**). Taken together, these findings suggest that NFκB and PI3K/Akt play essential roles in enhancement of TRAIL expression in CTLs by HG.

### Metformin and Vitamin D Protect Pancreatic Beta Cells From HG-CTL-Mediated Apoptosis

**Figure 5:**
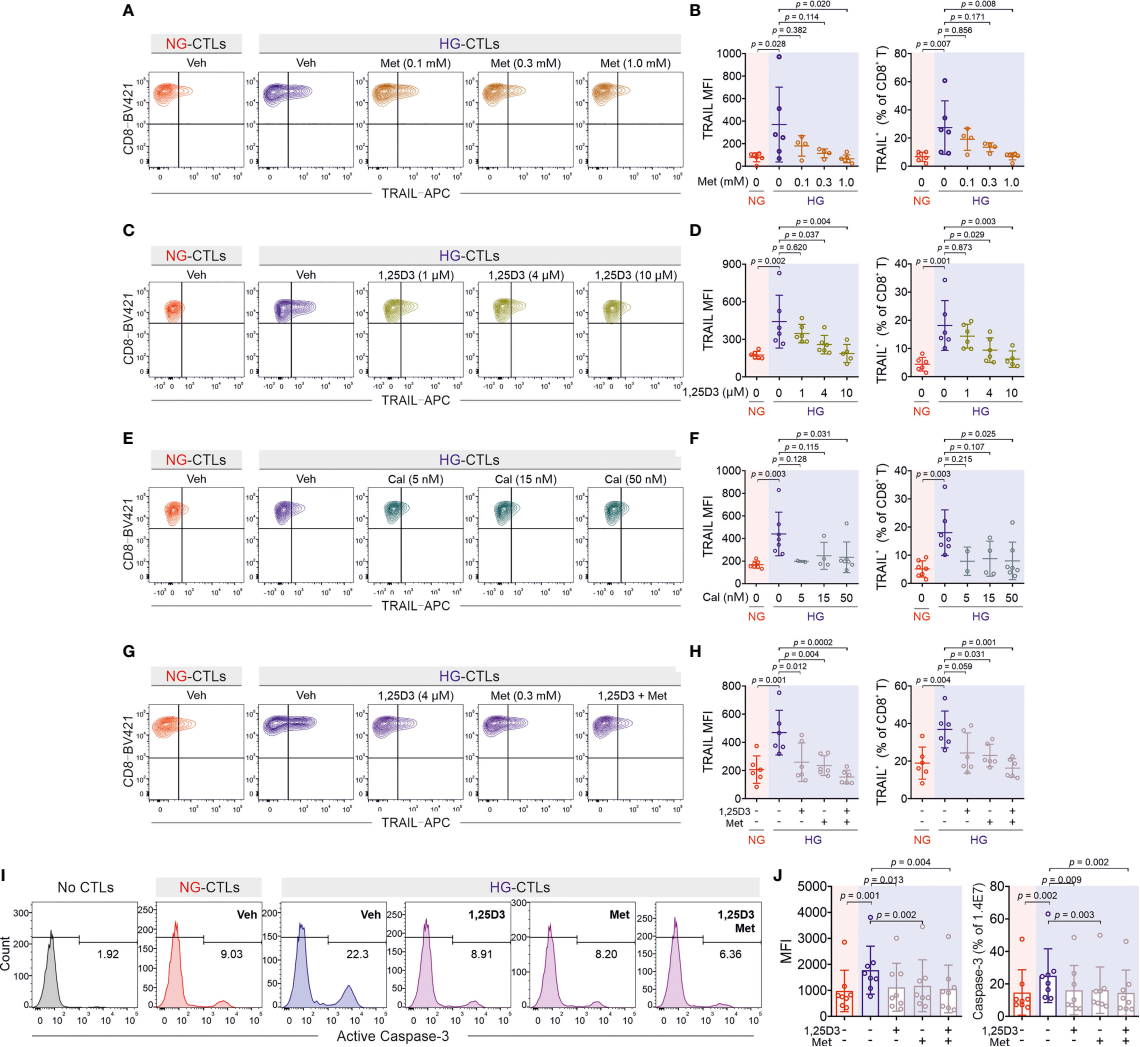
Treatment of metformin and vitamin D rescues TRAIL-mediated pancreatic beta cell destruction. Primary human CD8^+^ T cells were stimulated with CD3/CD28 beads for 3 days in presence of metformin (Met), 1,25D3, or calcipotriol (Cal) with indicated concentrations. TRAIL expression was analyzed by flow cytometry. **(A, B)** Treatment of metformin reduces HG-enhanced TRAIL expression in CD8^+^ T cells (n = 4-6 donors from four independent experiments). **(C, D)** Vitamin D regulates TRAIL expression (n = 5-6 donors from four independent experiments). **(E, F)** Enhancement of TRAIL expression by HG could be downregulated by calcipotriol (n = 2-7 donors from four independent experiments). **(G, H)** The combination of metformin and vitamin D synergistically inhibits HG-enhanced TRAIL expression (n = 6 donors from three independent experiments). **(I, J)** Metformin and vitamin D can rescue HG-enhanced CTL-mediated beta cell apoptosis. Primary human CD8^+^ T cells were stimulated with CD3/CD28 beads for 3 days in presence of metformin (Met, 300 µM) and/or Vitamin D (1,25D3, 4 µM). 1.4E7 beta cells were co-incubated with CTLs for 4 hours. Results are from 8 donors from six independent experiments. Data are shown as means ± SD. One-way ANOVA with Bonferroni’s multiple comparison test was applied for statistical analysis.

Since the enhancement of TRAIL levels on CTLs induced by HG leads to apoptosis of pancreatic beta cells as shown above (**Figure 2**), a decrease of TRAIL expression to normal levels should protect beta cells. We sought for possible therapeutical approaches to achieve this purpose. We first examined metformin, which is a widely applied first-line medication to treat type 2 diabetes. We stimulated primary human CD8^+^ T cells from healthy donors in medium containing NG or HG in presence or absence of metformin for three days. We found that TRAIL expression in HG-CTLs was significantly reduced by metformin in a dose-dependent manner (**Figures 5A, B**), down to the level of NG-CTLs at 1 mM (compare NG/vehicle and HG/Met 1 mM). These results suggest a putative protective role of metformin on TRAIL-mediated apoptosis of pancreatic beta cells.

Apart from metformin, vitamin D has been also linked to diabetes (29, 30), glucose metabolism (31, 32), protection of pancreatic beta cells (33), as well as HG-regulated cell functions (34). Therefore, we used 1,25-dihydroxy-vitamin D3 (1,25D3), the active form of vitamin D, and analyzed its impact on TRAIL expression. We found that HG-induced enhancement of TRAIL expression on CTLs was reduced by 1,25D3 in a dose-dependent manner (**Figures 5C, D**). Beside the naturally existing active form 1,25D3, the vitamin D analogue calcipotriol is widely used topically to treat psoriasis. Our results show that nanomolar concentrations of calcipotriol were sufficient to abolish HG-induced enhancement in TRAIL expression (**Figures 5E, F**). Our data suggest that activation of the vitamin D pathway can diminish HG-enhanced TRAIL expression on CTLs.

Since both metformin and vitamin D down-regulate HG-enhanced TRAIL expression, we tested whether they may function in a synergistic manner. We chose intermediate concentrations for metformin (0.3 mM) and 1,25D3 (4 µM), at which the down-regulation of TRAIL was modest in both cases (**Figures 5B, D**). Under this condition, we observed that metformin and 1,25D3 together further down-regulated TRAIL expression enhanced by HG (**Figures 5G, H**). Importantly, treatment with metformin or 1,25D3 did not affect T cell activation (**Figures S3A–D**) or viability (**Figure S3E**). These results indicate that metformin and vitamin D function in an additive manner to down-regulate HG-enhanced TRAIL expression on CTLs.

Our findings raise the important question whether metformin and/or vitamin D could protect pancreatic beta cells from HG-cultured CTLs by reducing their TRAIL expression. To address this question, we stimulated primary human CD8^+^ T cells in presence of metformin and/or 1,25D3. Subsequently CTLs were added to 1.4E7 beta cells without metformin and 1,25D3 to avoid the possible effect on beta cells per se. Analysis of 1.4E7 apoptosis at various time points shows that metformin, 1,25D3, or the combination of both reduced TRAIL-mediated beta cell apoptosis to the level of NG control cells (**Figures 5I, J**; **Figures S3F, G**). In summary, our results suggest that apoptosis of beta cells mediated by HG-induced TRAIL^high^ CTLs from healthy donors can be protected by metformin and/or vitamin D *in vitro*.

**Figure 6:**
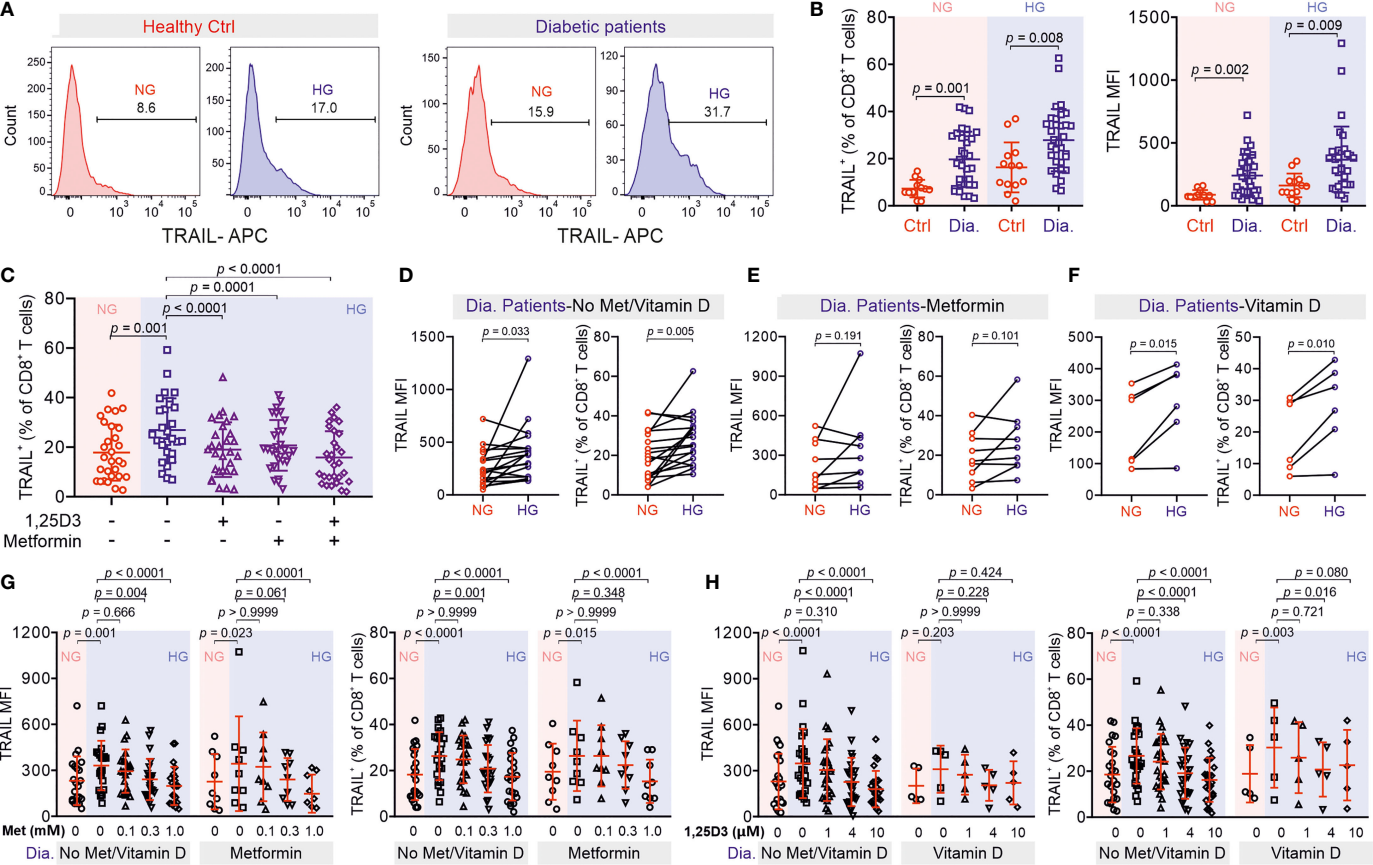
Treatment of metformin and vitamin D decreases TRAIL-expression in CTLs from diabetic patients. **(A, B)** TRAIL expression in CTLs from control and diabetic patients. PBMCs isolated from healthy individuals or diabetic patients were stimulated with CD3/CD28 beads in NG (5.6 mM, pink shade) or HG (25 mM, blue shade) medium for 3 days. Then the samples were stained with CD3, CD8 and TRAIL and analyzed by flow cytometry (Ctrl, n = 13 from thirteen independent experiments; diabetic patients, n = 33 from twenty-four independent experiments). One representative donor from either group is shown in **(A, C)** Metformin and vitamin D diminish enhanced TRAIL expression in diabetic CTLs. Diabetic CTLs from **(B)** were treated with met (300 µM) and/or 1,25D3 (4 µM) during bead-activation for 3 days (n = 29 from twenty-one independent experiments). **(D–H)** Diabetic patients from **(B)** were categorized into three groups based on whether metformin or vitamin D was taken. Data are represented as Mean ± SD and *p* values were assessed by two-tailed unpaired Student’s *t* test **(B)**, two-tailed paired Student’s *t* test **(D–F)**, one-way ANOVA **(C)**, or two-way ANOVA with Bonferroni’s multiple comparison test **(G, H)**.

To test the clinical relevance of metformin and vitamin D protection against TRAIL-induced beta cell apoptosis, we analyzed CTLs from patients diagnosed with diabetes. We found that after stimulation, the expression of TRAIL on diabetic CTLs was significantly higher compared to that of CTLs from healthy individuals, for both NG and HG conditions (**Figures 6A, B**). Remarkably, TRAIL expression of diabetic CTLs under the NG condition was already comparable to that of healthy CTLs in the HG condition (**Figure 6B**). TRAIL expression in diabetic CTLs did not differ between type 1 and type 2 diabetes (**Figures S4A, B**), or between males and females (**Figures S4C, D**), and did not correlate with the age (**Figures S4E, F**).

The correlations between TRAIL expression and diabetes prompted us to examine whether metformin and 1,25D3 can also regulate TRAIL expression in CTLs from diabetic patients. We found that TRAIL expression in diabetic CTLs was down-regulated by 1,25D3 (**Figure S5A**), calcipotriol (**Figure S5B**) or metformin (**Figure S5C**) in a dose-dependent manner. Together, metformin and 1,25D3 could even further suppress TRAIL expression in diabetic CTLs (**Figure 6C**). Interestingly, CTLs from patients who did not take metformin or vitamin D showed clear TRAIL enhancement in HG (**Figure 6D**), whereas CTLs from patients who took metformin did not show any difference between NG and HG (**Figure 6E**). CTLs from patients who took vitamin D exhibited only a moderate increase in TRAIL expression under HG condition (**Figure 6F**). For both the control group (no metformin and no vitamin D) and the metformin group, culturing CTLs from diabetic patients in presence of metformin in HG further down-regulated TRAIL expression (**Figure 6G**). Similarly, for the vitamin D group and the control group, culturing the diabetic CTLs with vitamin D in HG decreased TRAIL expression (**Figure 6H**). These findings suggest that HG-enhanced TRAIL expression on diabetic CTLs can be also down-regulated by metformin and vitamin D.

## Discussion

TRAIL is involved in the development of obesity and diabetes (11). In humans, the TRAIL family consists of four membrane receptors, TRAIL-R1, -R2, -R3 and -R4. Among them, TRAIL-R1 and -R2, also known as death receptor 4 (DR4) and 5 (DR5), are responsible for inducing TRAIL-mediated cell apoptosis, whereas TRAIL-R3 and -R4 are decoy receptors, which lack the cytoplasmic death domain. Thus, the ratio of death receptors over decoy receptors determines the sensitivity or efficacy of TRAIL-mediated cell apoptosis. TRAIL-R1 and -R2 are highly expressed in many tissues including kidney, heart, adipose tissue, and pancreas; in comparison, decoy receptors TRAIL-R3 and -R4 are not detectable in most of the tissues except for immune function-related organs/tissues (e.g. spleen, bone marrow, and lymph nodes) and pancreas (35). Infiltration of immune cells, especially CD8^+^ T cells, is positively correlated with the progression of diabetes and related complications (11). Therefore, it is likely that TRAIL^high^ CTLs induced by diabetic conditions not only contribute to destruction of pancreatic beta-cells in an antigen-independent manner but are also involved in attacking TRAIL-R1/-R2^high^ cells in different tissues resulting in undesirable complications. Our findings indicate that harnessing TRAIL expression in CTLs can therefore protect insulin-producing beta cells.

If down-regulation of TRAIL expression on CTLs could protect beta-cells, is progression of diabetes ameliorated by TRAIL deficiency? Feeding high-fat diet to a TRAIL/ApoE double knock-out strain significantly enhanced their fasting glucose compared to ApoE-/- or TRAIL-/- mice (36). Along this line, intraperitoneal injection of soluble TRAIL in mice fed with high-fat diet reduces beta-cell loss (37). An obvious question is why systemic blockade of TRAIL exacerbates diabetes? Compelling evidence show that TRAIL is an essential regulator to suppress the function of diabetogenic T cells, which recognize autoantigens on pancreatic beta-cells leading to their destruction. For example, injection of soluble DR5 (TRAIL-R2) in NOD mice elevates proliferation of GAD65-specific T cells (15). Along this line, blockade of TRAIL enhances proliferation of transferred diabetogenic T cells in NOD mice (37). Therefore, systematic blockade of TRAIL cannot protect pancreatic beta-cells.

In many cell types, glucose transporters (Gluts), especially Glut1, are up-regulated upon activation. For example, expression of Glut1 is elevated upon activation in murine CD4^+^ T cells and macrophages to favor glucose glycolysis over oxidative phosphorylation (38). In human T cells, expression of Glut1 is reported to be enhanced in CD3/CD28 bead-stimulated T cells compared to naive T cells (38). In comparison, our results how that the expression of Glut1 at the protein level is not altered by HG and even marginally down-regulated at the mRNA level. Our microarray data suggest that the expression levels of other Gluts are not influenced by HG, at least at the mRNA level. This indicates that in CTLs, glucose transporters are unlikely the target proteins responsive to HG to mediate HG-induced metabolic reprogramming.

Type I interferons (IFNs, e.g., IFN-α and IFN-β) can induce TRAIL expression on T cells (39). However, we could not detect differences in IFN-α and IFN-β expression by microarray analysis between CTLs cultured in NG and HG. This indicates that type I IFNs produced by CTLs are very unlikely to be involved in HG-enhanced TRAIL expression in CTLs. Interestingly, compelling evidence show that IFN-α plays a critical role in initiation of type 1 diabetes (40–42). In a mouse model, blockade of IFN-α signaling at prediabetic stage prevents beta-cells from destruction by CTLs (43). Therefore, we postulate that prior to clinical disease-resulted elevation of blood glucose, TRAIL expression on CTLs could be induced by IFN-α, which could also contribute to development of diabetes.

ROS is a byproduct of cellular metabolic redox reactions, which is closely related to progression of diabetes and its complications (44). In Th17 cells, a high glucose-induced autoimmune reaction is mediated by ROS originated from mitochondria (45). Our results show that for human CTLs, ROS is indeed involved in regulating HG-enhanced TRAIL expression, but cytosolic rather than mitochondrial ROS appears to be decisive. This suggests that Th17 cells and CTLs may differ in their regulatory mechanisms in response to HG. Interestingly, ROS can activate the PI3K/Akt pathway as well as the NFκB pathway (46, 47), which we also found to be essential for HG-enhanced TRAIL expression. Thus, in CTLs, ROS could act upstream of the PI3K/Akt/NFκB axis to regulate HG-enhanced TRAIL expression.

Vitamin D is closely related to insulin resistance and onset of diabetes (48). Vitamin D also has a profound impact on the immune system (49). In particular, expression of the vitamin D receptor induced by initial TCR signaling *via* p38 is essential for upregulation of phospholipase C gamma 1, which plays a pivotal role in T cell activation and Treg/Th17 differentiation (50, 51). In addition, vitamin D could also accelerate the transition of Th1 cells from pro-inflammatory to anti-inflammatory phase by epigenetic remodeling (52). Interestingly, vitamin D is involved in maintaining mitochondrial functions to optimize cellular redox conditions, thus protecting cells from oxidative stress-related damages (53). We postulate that vitamin D could diminish HG-enhanced TRAIL expression through reducing intracellular ROS production.

The impact of combined use of vitamin D and metformin has been investigated in various pathological scenarios. For example, combination of vitamin D and metformin shows a positive chemopreventive effect on colorectal cancer in rat and mouse models (12) and could be potentially effective to treat patients with polycystic ovary syndrome based on its effect on menstrual cycle (54). A recent study reported that in a type 2 diabetes mouse model, which cannot be controlled by metformin alone, additional vitamin D therapy improved insulin sensitivity in skeletal muscles (55). This finding supports our findings that combination of metformin and vitamin D could be beneficial to diabetic patients by reducing HG-enhanced expression of TRAIL in CTLs to protect beta cells from TRAIL-mediated apoptosis. In summary, we suggest a novel mechanism of CTL involvement in progression of diabetes, which establishes CTLs as a possible target for combined metformin and vitamin D therapy to protect pancreatic beta cells of diabetic patients, especially for the patients who still have sufficient numbers of beta-cells stay undestroyed.

## Data Availability Statement

The original contributions presented in the study are included in the article/**Supplementary Material**. Microarray data have been deposited in the ArrayExpress database at EMBL-EBI (www.ebi.ac.uk/arrayexpress) under accession number E-MTAB-11441. Further inquiries can be directed to the corresponding author.

## Ethics Statement

Research carried out for this study with material from healthy donors (leukocyte reduction system chambers from human blood donors), from diabetic patients and the healthy individuals is authorized by the local ethic committee (declaration from Ha 84/15, Prof. Dr. Rettig-Stürmer/Amendment M Hoth and Ha 84/19, GC, respectively). Protocols for the STZ-induced diabetes mouse model for this study is approved by the local regulatory authorities (Animal experiment approval 49/2019, LP). The animal experiments were performed according to local, national, and European Union ethical guidelines.

## Author Contributions

WY designed and performed most experiments and all the corresponding analyses if not mentioned otherwise. AD and VH analyzed microarray data. CD and MHa carried out microarray and EM helped interpret the results. FK, GC, and FL collected blood samples from diabetic patients and controls, and supervised the treatment of the patients. LS-B and DB performed seahorse assay and the analysis. GS carried out qRT-PCR. AY, RZ, and AK helped with flow cytometry. LP provided diabetic mice. MHo helped with data interpretation and provided critical feedback on all aspects of the project. BQ generated concepts, designed experiments, and wrote the manuscript. All authors contributed to the article and approved the submitted version.

## Funding

This project was funded by the Deutsche Forschungsgemeinschaft (SFB 1027 A2 to BQ, A11 to MHo, C3 and ZX to VH, TRR219 (M04) to LP), and HOMFOR2019 (to BQ). The flow cytometer was funded by DFG (GZ: INST 256/423-1 FUGG). LS-B and DB are funded by the FNR, respectively by the PRIDE program (PRIDE/11012546/NEXTIMMUNE) and the ATTRACT program (A14/BM/7632103).

## Conflict of Interest

The authors declare that the research was conducted in the absence of any commercial or financial relationships that could be construed as a potential conflict of interest.

## Publisher’s Note

All claims expressed in this article are solely those of the authors and do not necessarily represent those of their affiliated organizations, or those of the publisher, the editors and the reviewers. Any product that may be evaluated in this article, or claim that may be made by its manufacturer, is not guaranteed or endorsed by the publisher.
