# Supplementary material for: Unspecific CTL Killing Is Enhanced by High Glucose via TNF-Related Apoptosis-Inducing Ligand
Source: Front Immunol. 2022 Feb 21;13:831680. doi: 10.3389/fimmu.2022.831680 (PMC8899024; doi:10.3389/fimmu.2022.831680)
Supplement: Supplementary file 1 [file DataSheet_1.pdf]

Supplementary Material

Supplementary Figures

Figure S1. HG-induced TRAIL up-regulation in CD8$^+$ T cells enhances beta cell apoptosis.

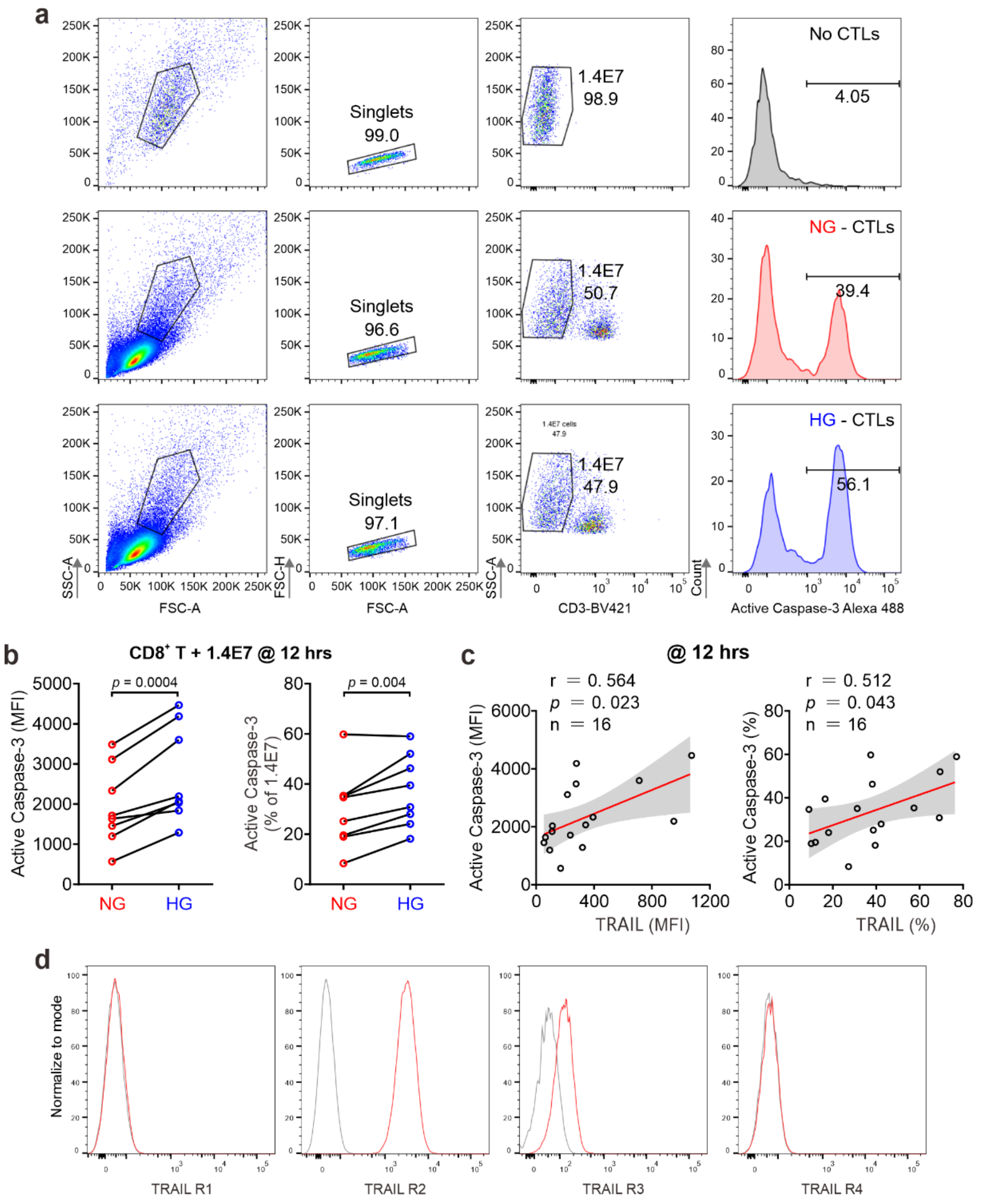

(**a**) Gating for apoptosis assay in 1.4E7 cells at 12 hours. (**b, c**) Apoptosis of pancreatic beta cell induced by CD8$^+$ T cells at 12 hours is shown in **b**. The correlation between TRAIL expression in CD8$^+$ T cells and 1.4E7 apoptosis of **b** is shown in **c**. Data were analyzed by two-tailed paired Student's *t* test (**b**), or Pearson's correlation coefficients (**c**). (**d**) Expression of TRAIL receptors in 1.4E7 beta cells. Gray: unstained samples.

Figure S2. Activation of mTOR and expression of glucose transporters in CTLs.

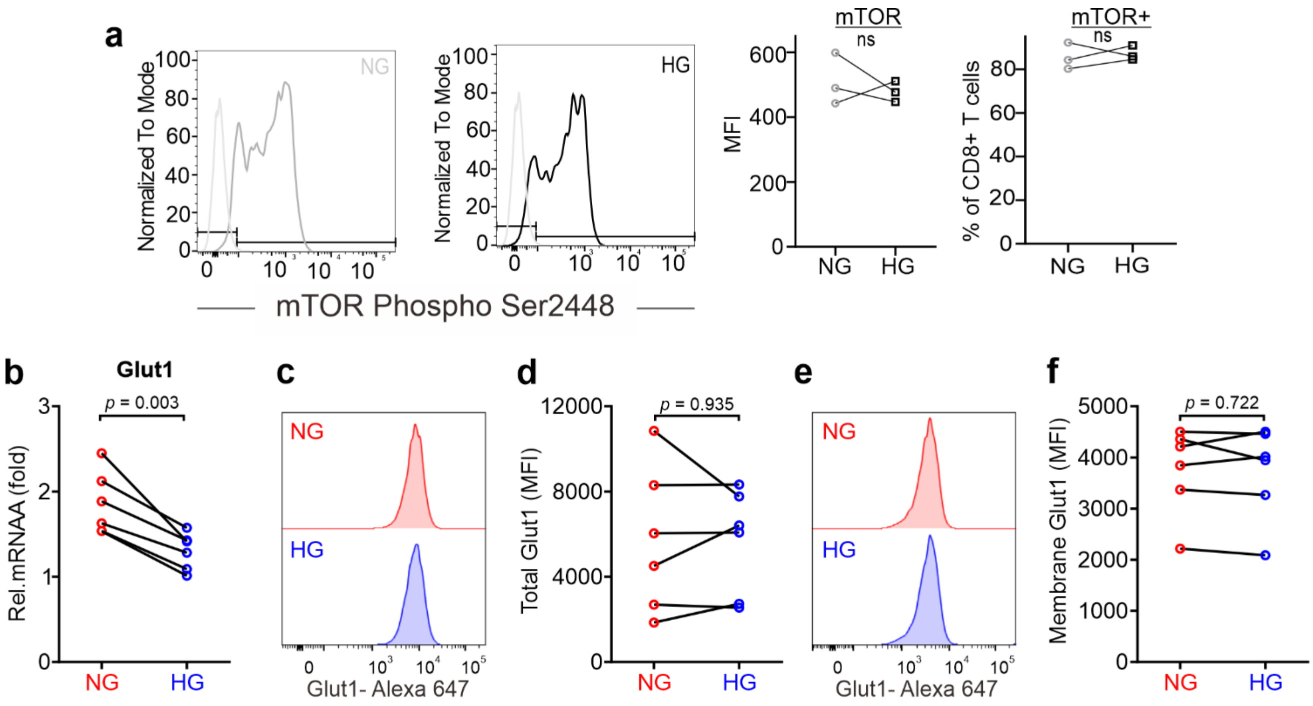

(**a**) Activity of mTOR in CTLs was not altered by HG. PBMCs were stimulated by CD3/CD28 beads and cultured in NG- or HG-medium for 3 days. Activity of mTOR was assessed by mTOR Phospho Ser2448. PBMCs were stained with anti-mTOR Phospho Ser2448, anti-CD3 and anti-CD8 antibody. $CD3^{+}CD8^{+}$ cells were gated for analysis. MFI: mean fluorescence intensity. (**b**) Transcriptional levels of Glut1 in CTLs were quantified by qRT-PCR. (**c-f**) Glut1 expression in CTLs in total (**c**, **d**) or on the surface (**e**, **f**) was determined by flow cytometry. Data were analyzed by two-tailed paired Student's *t* test (**b**, **d**, **f**). Results are from 3 donors two independent experiments (**a**), 6 donors from three (**b**, **f**) or four (**d**) independent experiments.

Figure S3. Metformin and vitamin D does not alter activation or viability of CTLs.

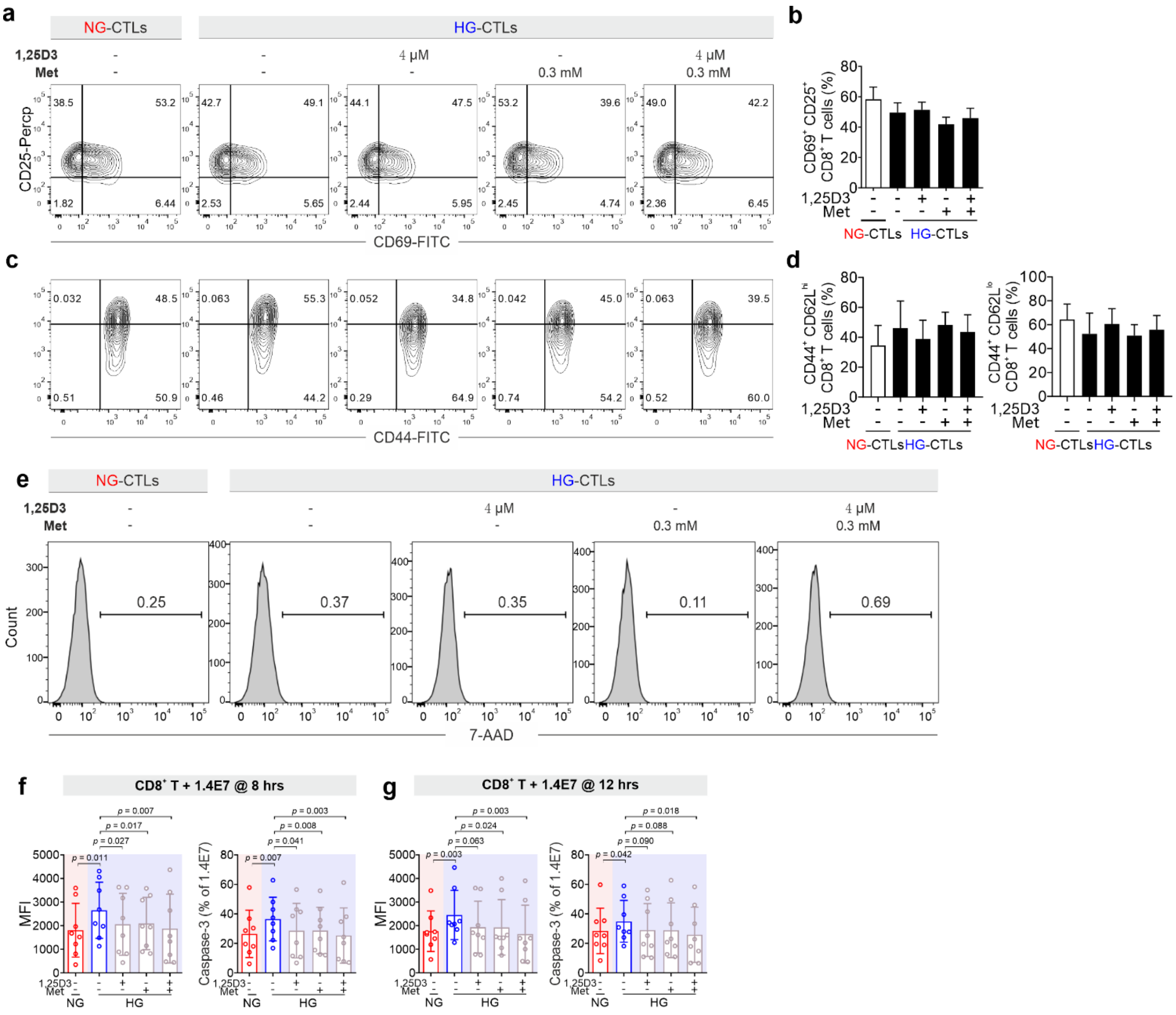

Primary human CD8⁺ T cells were stimulated with CD3/CD28 beads in NG (5.6 mM) or HG (25 mM) for 3 days in presence with 1,25D3 (4 µM) and/or metformin (300 µM). (**a-d**) Activation of CD8⁺ T cells (n = 3-4 donors). Results are represented as Mean ± SD. (**e**) Apoptosis of CD8⁺ T cells. (**f**, **g**) Met and 1,25D3 can rescue HG-enhanced beta cell apoptosis by CD8⁺ T cells. Apoptosis of 1.4E7 beta cells was determined by the activity of Caspase-3 at 8 hour (**f**) and 12 hours (**g**) (n = 8 donors) from six (**g**) or seven (**f**) independent experiments. Data were analyzed by one-way ANOVA with Bonferroni's multiple comparison test.

Figure S4. Treatment with met or 1,25D3 can abolish HG-enhanced TRAIL expression in CD8+ T cells of diabetic patients.

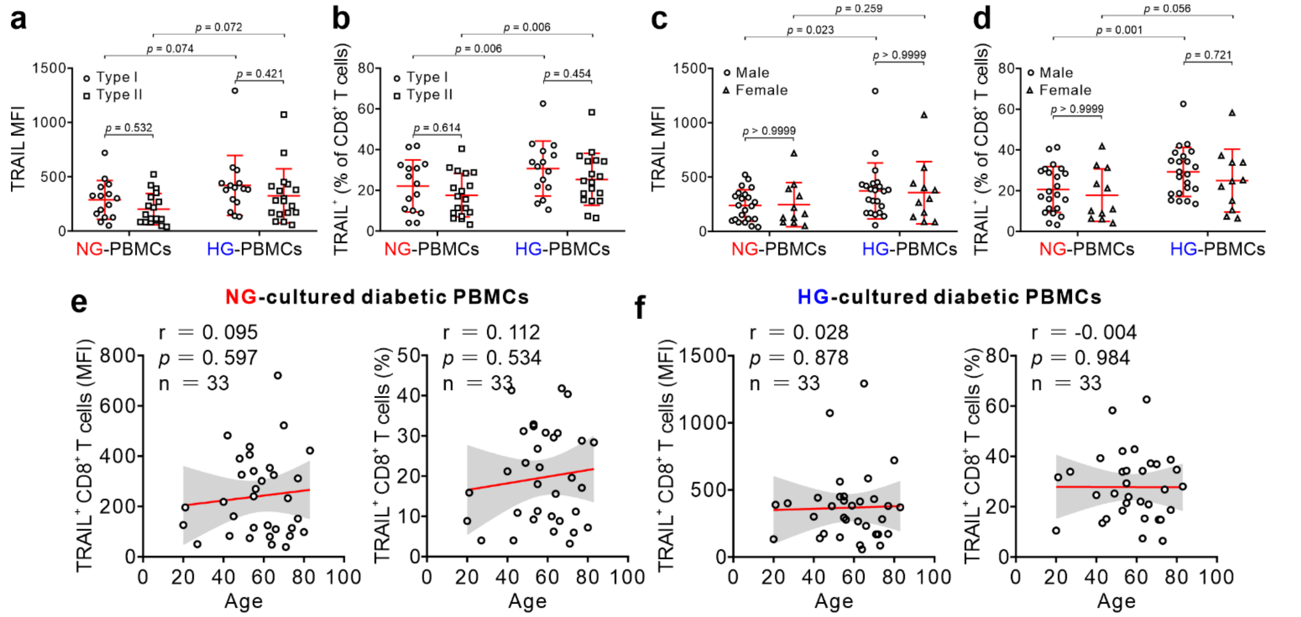

TRAIL expression is not correlated with types (**a**, **b**), gender (**c**, **d**) or age (**e**, **f**). PBMCs from diabetic patients same as in Fig. 5b. Results were represented as Mean ± SD. Data were analyzed by two-way ANOVA with Bonferroni's multiple comparison test (**a-d**), or Pearson's correlation coefficients (**e**, **f**).

Figure S5. Impact of Metformin or vitamin D pathway on HG-enhanced TRAIL expression in CD8$^+$ T cells.

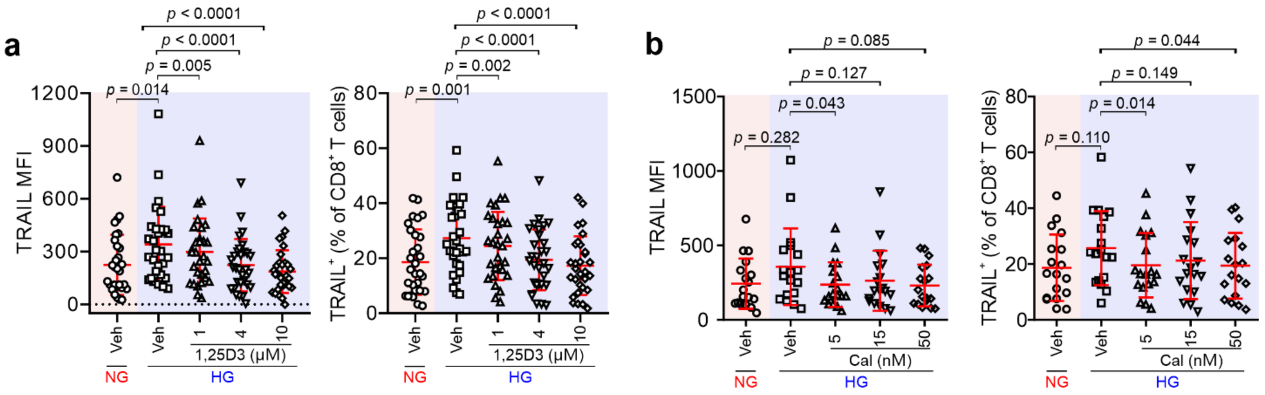

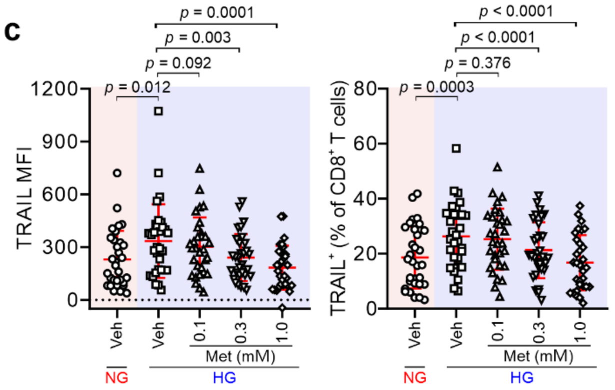

PBMCs from diabetic patients were stimulated with CD3/CD28 beads in presence of vitamin D (1,25D3, **a**), calcipotriol (Cal, **b**) metformin (Met, **c**) for 3 days. TRAIL expression was determined by flow cytometry. Results are represented as Mean ± SD (1,25D3 n = 30; Cal n = 18; Met n = 31 from 21, 14 or 23 independent experiments). Data were analyzed by one-way ANOVA with Bonferroni's multiple comparison test.

Supplementary table legends

Table S1. Normalized gene expression. Microarray gene expression data were processed with NormExp background correction and quantile normalization. The control probes were removed, as well as probes that were not expressed in at least two out of 12 samples. Remaining probes were annotated with gene symbols and entrez identifiers, according to the specifications of the microarray platform (Agilent design number 026652). Results were from 12 samples of 6 donors.

Table S2. GO term enrichment analysis for metabolism. Enriched Gene Ontology (GO) terms for pairwise comparisons between conditions. GO term enrichment analysis was performed with the goana function from the limma R-package. Each term was annotated with its respective up- and down-regulated genes. Go terms contain *metabolism* are shown.

Table S3. GO term enrichment analysis for ROS. Enriched Gene Ontology (GO) terms for pairwise comparisons between conditions. GO term enrichment analysis was performed with the goana function from the limma R-package. Each term was annotated with its respective up- and down-regulated genes. Go terms contain *reactive oxygen spieces* are shown.

Table S4. KEGG pathway enrichment analysis. Enriched KEGG pathways for each pairwise comparison between conditions. Pathway enrichment analysis was performed with the kegga function from the limma R-package. Each pathway was annotated with its respective up- and down-regulated genes.
